# Understanding the Significance of the Hypothalamic Nature of the Subthalamic Nucleus

**DOI:** 10.1523/ENEURO.0116-21.2021

**Published:** 2021-10-04

**Authors:** Marie Barbier, Pierre-Yves Risold

**Affiliations:** 1 Department of Psychiatry, Seaver Autism Center for Research and Treatment, Department of Neuroscience, Friedman Brain Institute, New York, NY 10029; 2 Neurosciences Intégratives et Cliniques EA481, Université de Bourgogne Franche-Comté, Besançon 25000, France

**Keywords:** behavior, hypothalamus, neuroanatomy, system neuroscience

## Abstract

The subthalamic nucleus (STN) is an essential component of the basal ganglia and has long been considered to be a part of the ventral thalamus. However, recent neurodevelopmental data indicated that this nucleus is of hypothalamic origin which is now commonly acknowledged. In this work, we aimed to verify whether the inclusion of the STN in the hypothalamus could influence the way we understand and conduct research on the organization of the whole ventral and posterior diencephalon. Developmental and neurochemical data indicate that the STN is part of a larger glutamatergic posterior hypothalamic region that includes the premammillary and mammillary nuclei. The main anatomic characteristic common to this region involves the convergent cortical and pallidal projections that it receives, which is based on the model of the hyperdirect and indirect pathways to the STN. This whole posterior hypothalamic region is then integrated into distinct functional networks that interact with the ventral mesencephalon to adjust behavior depending on external and internal contexts.

## Significance Statement

In this work, we suggest that networks between the telencephalon, including cerebral cortex and basal nuclei, with the whole posterior hypothalamus, including the subthalamic nucleus (STN), posterior lateral hypothalamic, premammillary, and mammillary nuclei, are built along topographically organized pathways that parallel the hyperdirect and indirect pathways that are characteristic of the basal ganglia network. This suggests a high degree of organizational convergence between the basal ganglia and longitudinal hypothalamic networks to control the expression of behavioral responses adapted to external and internal cues.

## Introduction

**Figure 1.**
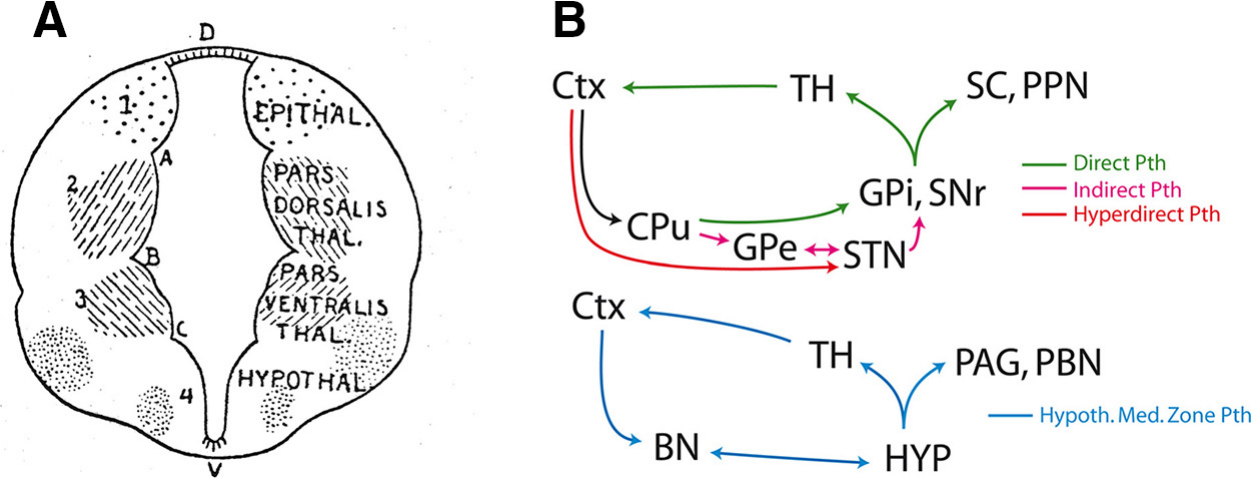
Organization of the diencephalon and of prosencephalic networks. ***A***, Proposed divisions of the diencephalon by Herrick (Herrick, 1910). ***B***, Model of circuitries involving the basal ganglia (top) and the medial zone nuclei of the hypothalamus (bottom). Both involve loop pathways with the thalamus and the cortex. The descending projections of the basal ganglia are classically divided into direct, indirect, and hyperdirect pathways. Such pathways for the medial zone nuclei of the hypothalamus have not yet been identified. BN: basal nuclei; CPu: caudoputamen nucleus; Ctx: cerebral cortex; GPe: globus pallidus, external part; GPi: globus pallidus, internal part; HYP: hypothalamus; PAG: periaqueductal gray; PBN: parabrachial nuclei; PPN: pedunculopontine nucleus; SC: superior colliculus; SNr: substantia nigra; reticular part; STN: subthalamic nucleus; TH: thalamus.

Initially the whole ventral diencephalon was included in a region named “regio subthalamica” by Forel (Forel, 1877) or “hypothalamus” by Wilhelm His (His, 1893). However, Herrick (Herrick, 1910) made the distinction between the hypothalamus proper, which covers a large collection of nuclei and areas within the ventral margin of the diencephalon, and the ventral thalamus, which essentially comprises the reticular thalamic nucleus, the zona incerta and the subthalamic nucleus (STN; Fig. 1*A*). This organization model was largely adopted until the end of the 20th century as it seemed to agree with functional differences: the hypothalamus is involved in the control of neuroendocrine/autonomic responses as well as the expression of instinctive behaviors, while the ventral thalamus participates in higher cognitive processes or voluntary motor actions by mediating cortico-thalamic interactions or as part of the basal ganglia network. However, in the late 20th century, the borders as well as the internal organization of these brain regions were strongly debated once again. The former consensus that both the ventral thalamus and the hypothalamus belong to the ventral diencephalic vesicle was shaken by evidence that both regions are best regarded as rostral rather than ventral to the thalamus (Puelles and Rubenstein, 2015; Puelles et al., 2019). The borders between the hypothalamus and ventral thalamus were disputed yet again. For example, in 1980, it was believed that the STN undeniably belonged to the ventral thalamus; however, it is now considered to be a part of the hypothalamus (Altman and Bayer, 1986; Swanson, 2004, 2012). Furthermore, while the STN ventral thalamic identity was being challenged, organizational analogies between the basal ganglia and the hypothalamic networks were also recognized. Indeed, the systematic study of hypothalamic medial zone nuclei connections led to the conclusion that these nuclei are entangled in loop circuits with the thalamus, cerebral cortex and cerebral nuclei that parallel similar loops that are representative of the basal ganglia network in which the STN is integrated (Fig. 1*B*; Risold et al., 1994, 1997; Risold and Swanson, 1995, 1996; Swanson, 2000, 2012).

Unfortunately, this dramatic increase in our knowledge about the development and anatomy of the forebrain has not yet led to a new accepted view of the organization of the forebrain that can be shared with a general audience. In brief, neuroanatomists and developmentalists know that the former concepts of the forebrain organization are not in tune with our actual knowledge; however, a new and accepted schema has struggled to emerge and such changes as the anatomic identity of the STN may be viewed by many other neuroscientists as merely a matter of academic discussion, without any tangible consequences. In contrast, it is now appropriate to think about the implication of the STN having a hypothalamic identity as this will profoundly influence our understanding of the organization of the posterior hypothalamus and thus the hypothalamus and forebrain altogether.

In this work, we analyze available data in the literature about the development, connectivity, and functions of the STN and of the neighboring posterior hypothalamic cell groups. We demonstrate that a specific glutamatergic posterior hypothalamic region that comprises nuclei from the STN to the mammillary body (MBO), receives convergent cortical and pallidal inputs from the telencephalon and is involved, along the striatally targeted ventral mesencephalon, in the coordinated control of the behavioral response of the individual.

## The STN Belongs to the Posterior Hypothalamus

**Figure 2.**
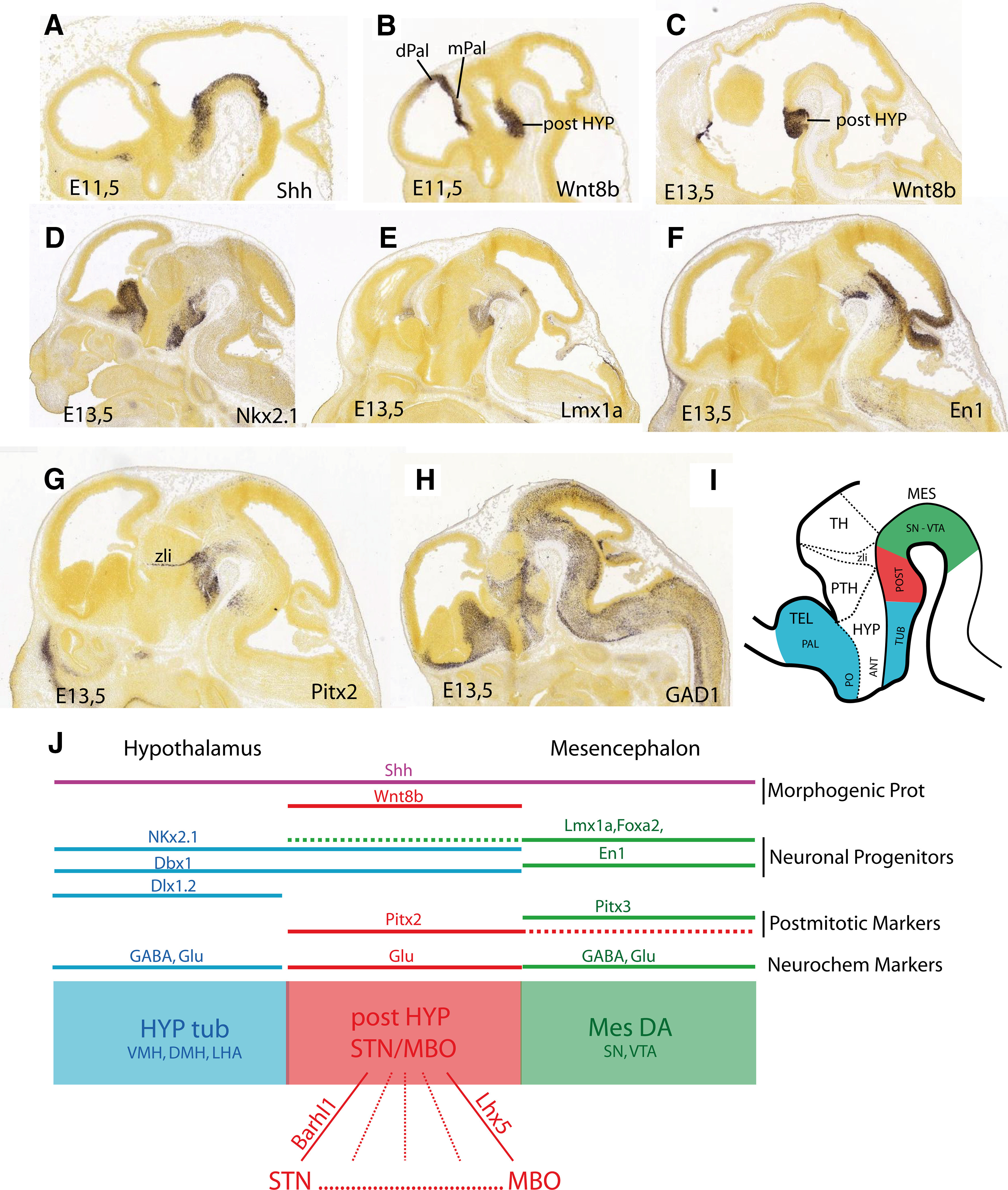
Development of the posterior hypothalamus. ***A–C***, Pictures reprinted from the Allen Brain Institute (image credit: Allen Institute; 2020 Allen Institute for Brain Science; Allen Brain Atlas: Mouse Brain; available from http://mouse.brain-map.org/experiment/show/100092704, http://mouse.brain-map.org/experiment/show/100029214, and http://mouse.brain-map.org/experiment/show/100030632) and illustrating the distribution of genes coding for the morphogenic proteins *Shh* and *Wnt8b* on sagittal sections of embryonic brains (embryonic stages 11.5 or 13.5). ***D–F***, Pictures reprinted from the Allen Brain Institute (image credit: Allen Institute; available from: http://mouse.brain-map.org/experiment/show/100093267, http://mouse.brain-map.org/experiment/show/100076539, and http://mouse.brain-map.org/experiment/show/100030677) and illustrating the distribution of the neuronal progenitors *Nkx2.1*, *Lmx1a*, and *En1* on sagittal sections of the embryonic mouse brain. ***G–H***, Pictures reprinted from the Allen Brain Institute (image credit: Allen Institute; available from: http://mouse.brain-map.org/experiment/show/100026263 and http://mouse.brain-map.org/experiment/show/100076531) to illustrate the embryonic distribution of the postmitotic transcription factor *Pitx2* and the enzyme GAD. ***I***, Line drawing summarizing the division of the embryonic prosencephalon and the distribution of *Nkx2.1* (blue and red) and *Lmx1a* (green and red). ***J***, Diagram illustrating the distribution of transcription factors involved in the differentiation of the posterior hypothalamus. The development of the ventral mesencephalon/posterior hypothalamic continuum depends on the action of morphogenetic proteins such as SHH. However, the expression domain of *Wnt8b* is specific of the posterior hypothalamus. The posterior hypothalamic anlage is characterized by the expression of hypothalamic (*Nkx2.1*, *Dbx1*) and mesencephalic (*Lmx1a*, *Foxa2*) neuronal progenitor genes. Some postmitotic transcription factors are also common to the mesencephalon, but then each nucleus of the posterior hypothalamus necessitates the action of specific transcription factors such as *Barhl1* for the STN or *Lhx5* for the MBO. Finally, the posterior hypothalamic region is massively glutamatergic while adjacent territories contain a mix of GABAergic and glutamatergic neurons. ANT: presumptive anterior area of the hypothalamus; DMH: dorsomedial nucleus hypothalamus; Glu: glutamate; dPal: dorsal pallium; mPal: medial pallium; HYP: hypothalamus; LHA: lateral hypothalamic area; MBO: mammillary nuclei; MES: mesencephalon; MesDA: DAergic ventral mesencephalon: mPal: medial pallium; PAL: pallidum; PO: presumptive preoptic area; POST: presumptive posterior hypothalamic area; PTH: prethalamus (ventral thalamus); SN: substantia nigra; STN: subthalamic nucleus; TEL: telencephalon; TH: thalamus; TUB: presumptive tuberal hypothalamic area; VMH: ventromedial hypothalamic nucleus hypothalamus; VTA: ventral tegmental area; zli: zona limitans intrathalamica.

**Figure 3.**
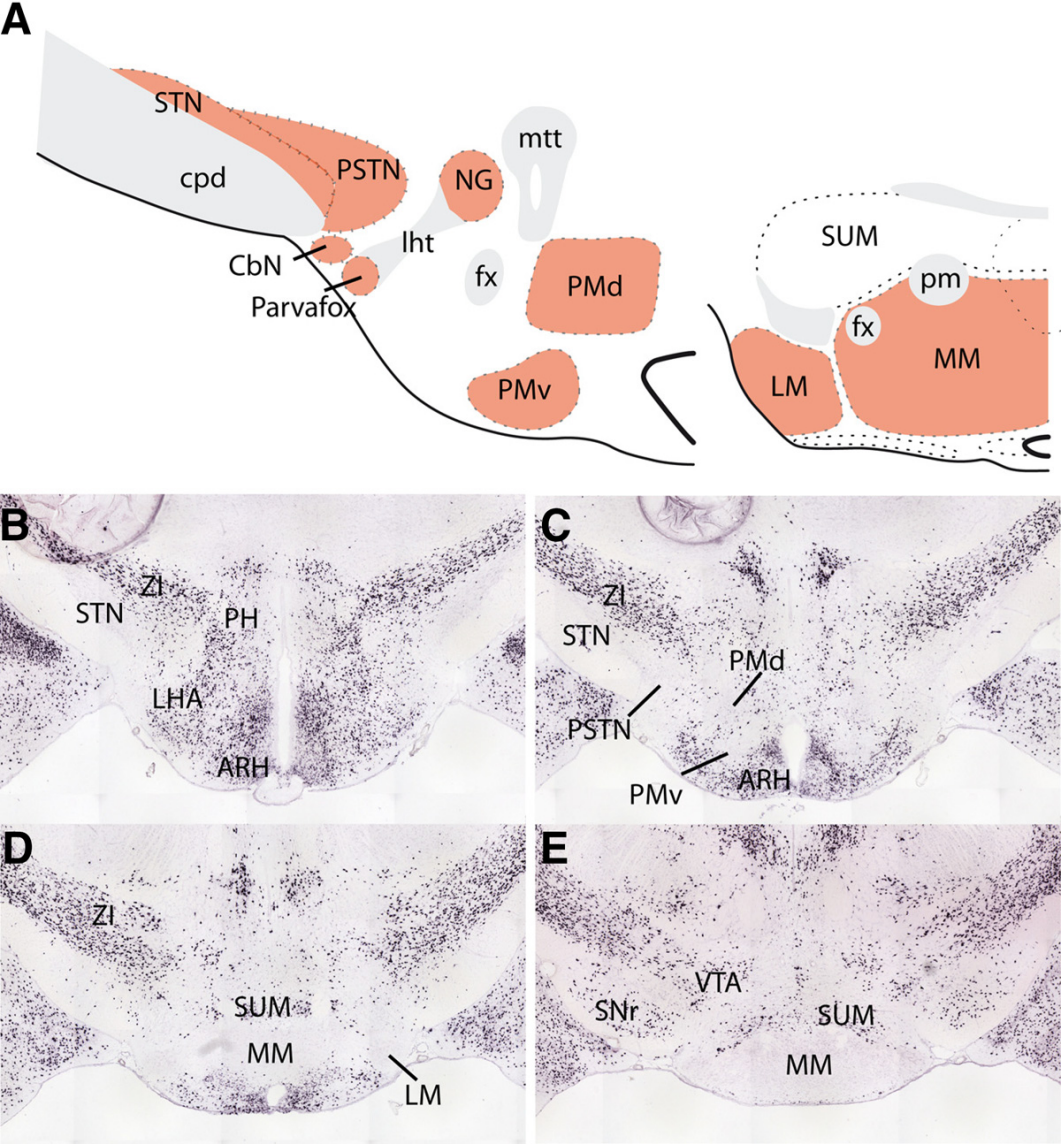
Architecture of the glutamaergic posterior hypothalamus. ***A***, Line drawing to illustrate the nuclear parcellation of the glutamatergic posterior hypothalamus in the rat. The pink nuclei are glutamatergic. ***B–E***, Pictures reprinted from the Allen Brain Institute (image credit: Allen Institute; 2020 Allen Institute for Brain Science; Allen Brain Atlas: Mouse Brain; available from http://mouse.brain-map.org/experiment/show/79591669) to illustrate the distribution of GAD2 in the posterior hypothalamus of the mouse. ARH: arcuate nucleus of the hypothalamus; CbN: calbindin nucleus; cpd: cerebral peduncle; fx: fornix; LHA: lateral hypothalamic area hypothalamus; lht: lateral hypothalamic tract; LM: lateral mammillary nucleus; MM: medial mammillary nucleus; mtt: mammillothalamic tract; NG: nucleus gemini; PH: posterior hypothalamic nucleus; pm: principal mammillary tract; PMd: dorsal premammillary nucleus; PMv: ventral premammillary nucleus; PSTN: para-STN; SNr: substantia nigra, reticular part; STN: subthalamic nucleus; SUM: supramammillary nucleus; VTA: ventral tegmental area; ZI: zona incerta.

The STN was first named after its discoverer, the French neurologist Jules Bernard Luys (1828–1897), before receiving its definitive appellation as the “nucleus subthalamicus” (in Altman and Bayer, 1986). A hypothalamic identity for the STN was suggested by Rose (Rose, 1942) and Kuhlenbeck (Kuhlenbeck, 1973) in the 20th century, against the dominant perception that this region is located within the ventral thalamus. However, to the best of our knowledge, Altmann and Bayer (Altman and Bayer, 1986) were the first to show that the STN is generated within the caudal hypothalamic anlage. In a comprehensive study of the development of the hypothalamus, these authors showed that “postmitotic subthalamic neurons migrate by a semicircular route from the anterodorsal mammillary recess neuroepithelium” following an outside-in gradient, as classically described for the hypothalamus. Therefore, following the work of Altmann and Bayer, it can be stated that neurons of the STN are generated in a region that adjoins the premammillary (PM) and mammillary nuclei and, therefore, the STN is a part of the posterior hypothalamus. From the 1990s to the present day, the analysis of the distribution and action of dozens of developmental genes, many of which encode morphogenic proteins or transcription factors, has resulted in a better understanding of the precise molecular orchestration that drives brain patterning and neurogenesis (Puelles and Rubenstein, 1993, 2015; Rubenstein et al., 1994; Rubenstein and Puelles, 1994; Alvarez-Bolado et al., 1995; Shimogori et al., 2010; Diez-Roux et al., 2011; Moreno and González, 2011; Puelles et al., 2013). Therefore, information about the mechanism that governs the formation of the posterior hypothalamus is slowly emerging (Bedont et al., 2015; Kim et al., 2020). Based on the current literature, it can be stated that the initial processes involved in the differentiation of the posterior hypothalamic and the ventral mesencephalic anlagen depend on the diffusion of morphogenic proteins that drive the expression of transcription factors through the mesodiencephalic floorplate (Fig. 2; Alvarez-Bolado et al., 2012; Bedont et al., 2015). While the processes involved in the interactions between these proteins are not yet fully clear, the early distribution of these molecules delimits three domains (Alvarez-Bolado et al., 2012; Bedont et al., 2015; Nouri and Awatramani, 2017). (1) Above the mesencephalic flexure, the ventral mesencephalic domain produces dopaminergic (DAergic) neurons in the substantia nigra (SN)/ventral tegmental area (VTA). (2) The ventral floor plate of the diencephalon is lined by a postoptic hypothalamic domain that is often referred to as the tuberal hypothalamus and in which the ventromedial hypothalamic nucleus (VMH), dorsomedial hypothalamic nucleus (DMH) and tuberal lateral hypothalamic area (LHA) are produced. (3) Between the mesencephalic and tuberal hypothalamic anlagen, we find the posterior hypothalamic domain. This domain produces the STN, parasubthalamic nucleus (PSTN), calbindin nucleus (CbN), Parvafox nucleus, Gemini nucleus, ventral PM (PMv), dorsal PM (PMd), and MBO (Fig. 3). These three domains require the expression of the morphogenic protein sonic hedgehog (SHH). However, the posterior hypothalamic anlage is also characterized by the specific expression of *Wnt8b* (Fig. 2). The role of the expression of this gene is unknown, but an interplay between *Shh* and *Wnt8b* has been observed in the patterning of the dorsomedial pallium which is another region showing intense *Wnt8b* expression that gives rise to cortical areas that, as we will see, are connected to the posterior hypothalamus in the mature brain. This posterior hypothalamic domain also expresses neuronal progenitor markers such as the transcription factors *Nkx2.1* and *Dbx1* which play important roles in hypothalamic patterning and are expressed in the tuberal hypothalamus (Fig. 2). The expression of *Nkx2.1* is restricted to two regions of the prosencephalon (Rubenstein and Puelles, 1994; Kimura et al., 1996; Sussel et al., 1999; Flandin et al., 2010; Moreno and González, 2011; Alvarez-Bolado et al., 2012; Magno et al., 2017): a large basal telencephalic zone encompassing the pallidum and the preoptic area, and a postoptic territory that includes the tuberal and posterior hypothalamus. Since *Nkx2.1* is expressed throughout most of the hypothalamus except a restricted anterior region between the preoptic and postoptic hypothalamus, it is often considered a hypothalamic marker. Experimental silencing of the *Nkx2.1* gene, critically perturbs the formation of the hypothalamus leading to a reduction in the size of many tuberal structures such as the VMH, DMH, or LHA, and ablation of the mammillary/premammillary structures as well as the STN (Kimura et al., 1996; Kim et al., 2020). *Dbx1* is required for the differentiation of many hypothalamic cell types in both the tuberal and the posterior hypothalamus (Sokolowski et al., 2016; Nouri and Awatramani, 2017; Alvarez-Bolado, 2019). Therefore, according to the early distribution and functions of *Nkx2.1* and *Dbx1*, the region that gives birth to the STN and MBO is hypothalamic in nature. However, recent studies also point toward intriguing relationships between mesencephalic and posterior hypothalamic neuronal lineages. As the grafting of DAergic neurons produced from embryonic or induced pluripotent stem cells is a promising field of research for the development of treatments for Parkinson’s disease, much attention has been focused on the genetic mechanisms involved in the differentiation of these neurons (Kirkeby et al., 2017). Therefore, many of the progenitor and postmitotic markers of DAergic neurons have been identified. Interestingly, most of the currently known DAergic progenitor markers, including *Lmx1a* and *Foxa2*, among others, are also expressed rostrally to the mesencephalic anlage into the posterior hypothalamus, but not into the tuberal hypothalamic domain (Kee et al., 2017; Nouri and Awatramani, 2017). Nouri and Awatramani (Nouri and Awatramani, 2017) dissected the distribution of *Lmx1a* and *Foxa2* in the posterior hypothalamus. They showed intense expression of the two progenitor markers in STN, PSTN, and PMv neurons coexpressing *Dbx1*. The close relationship between the cell lineage of the posterior hypothalamus and MES-DA may also be reflected by the expression of the DA transporter (DAT) in adult PMv neurons (Stagkourakis et al., 2018), whereas this protein is otherwise found only in DAergic neurons throughout the midbrain/forebrain (Ciliax et al., 1995). In wild-type embryos, the rostral boundary of *En1* expression in the ventral mesencephalon abuts the expression domain of *Dbx1* in the posterior hypothalamus (Nouri and Awatramani, 2017). It is suspected that some corepressive interactions take place between these two transcription factors which are probably important for maintaining the respective identity of the ventral mesencephalon and of the posterior hypothalamus (Nouri and Awatramani, 2017). Indeed, the forced expression of *En1* in the posterior hypothalamic region induces the ectopic differentiation of DAergic neurons scattered in the mammillary region (Kee et al., 2017).

In addition to early progenitor markers, postmitotic transcription factors such as *Pitx2* are also necessary for the development of both the ventral mesencephalon and the posterior hypothalamus. In the posterior hypothalamus, *Pitx2* plays a determinant role in the migration of STN neurons or the establishment of the mammillothalamic tract and is still expressed in the entire posterior hypothalamus of adult mice (Smidt et al., 2000; Skidmore et al., 2012; Waite et al., 2013). However, most postmitotic DAergic neuron markers such as *Pitx3* are not found in the posterior hypothalamus (Kee et al., 2017). Each nucleus of the posterior hypothalamus is otherwise characterized by a specific combination of transcription factors, such as *Barlh1* for the STN (Kee et al., 2017) or *Lhx5* and *Fkh5* for the MBO (Wehr et al., 1997; Heide et al., 2015; Miquelajáuregui et al., 2015), but the lineages of most cell types constituting this region still require investigation.

An important neurochemical feature needs to be stressed here as it characterizes most of the posterior hypothalamic region and has important functional consequences: posterior hypothalamic structures are mostly glutamatergic while abundant GABAergic neurons can be found in the adjacent tuberal hypothalamus (DMH, LHA), zona incerta and ventral mesencephalon (SN, VTA). In the embryonic posterior hypothalamic domain, the lack of *Dlx* and *Gad* gene expression distinguishes the posterior hypothalamus from adjacent structures (Puelles et al., 2012, 2013; Figs. 2, 3). The *Dlx* genes code for transcription factors that are responsible for orienting differentiating neurons toward a GABAergic phenotype (Lindtner et al., 2019). The glutamic acid decarboxylase (GAD) enzyme is necessary for the synthesis of GABA (Esclapez et al., 1993; McDonald and Augustine, 1993). In the adult brain, GABAergic cells are present in the posterior hypothalamic nucleus and the capsule of the PMv that are close to the tuberal hypothalamus or in the supramammillary nucleus that abuts the VTA (Esclapez et al., 1993). However, the nuclei that form the core of this region, namely, the STN, PSTN, Parvafox, Gemini nucleus, core of the PMv, PMd, and MBO are massively glutamatergic and contain very few or no GABAergic cells (Fig. 3).

Therefore, the STN differentiates within a specific anlage that also produces premammillary and mammillary nuclei. The MBO was already included in the hypothalamus by His (His, 1893), and some of the genes that are necessary for the differentiation of this posterior hypothalamic region are emblematic hypothalamic markers. However, this region also requires the expression of progenitor markers that are needed for the development of the ventral mesencephalon and they display a specific feature by being massively glutamatergic.

## Convergence of Cortical and Pallidal Projections into the Posterior Hypothalamus

As the STN shares clear developmental and neurochemical features with premammillary and mammillary nuclei, the appraisal of comparable anatomic traits is legitimate. Historically, the circuit involving the MBO was first described by James Papez in 1937 (Papez, 1995). This circuit involves a strong hippocampal input that reaches the MBO through the fornix, a very conspicuous tract that longitudinally crosses the entire anterior and postoptic hypothalamus. By comparison, the STN is targeted by isocortical projections that constitute the hyperdirect pathway of the basal ganglia. It also receives abundant projections from the pallidum in the basal telencephalon, constituting the well-described indirect pathway of the basal ganglia. Therefore, the cortex and the pallidum could be important sources of afferences that drive the activity of neurons in this region.

### Cortical afferences or hyperdirect pathways

#### The basal ganglia hyperdirect pathways

The hyperdirect pathway of the basal ganglia is still the subject of regularly published anatomic articles using classic tract tracing or modern tractography (Chen et al., 2020; Temiz et al., 2020). Observations in humans, primates and rodents are concordant, and the STN can be subdivided into three domains partially depending on the origin of the cortical input. Many authors recognize a large dorsolateral motor, a ventral associative and a medial “limbic” sector (Parent and Hazrati, 1995a; Emmi et al., 2020). This tripartite organization of the STN is debated because no obvious boundaries can be traced within the nucleus and projections from the telencephalon often overlap. Nevertheless, this points toward a topographical organization in the telencephalic (including cortical) afferences to the nucleus. The latest studies conducted in humans and primates extended the concept of the hyperdirect pathway to include the LHA that is medially adjacent to the STN (Haynes and Haber, 2013; Temiz et al., 2020). This region is referred to as the “medial subthalamic region” in primates and humans, and it receives projections from the ventral medial prefrontal, entorhinal and insular cortices that do not innervate the STN proper. Therefore, in primates including humans, the STN receives isocortical projections while periallocortical areas such as the ventral medial prefrontal and insular areas, target LHA regions that are medially adjacent to the STN. In rodents, a similar observation was made, but, in contrast to that in primates, the LHA nuclei medially adjacent to the STN are well characterized (Barbier et al., 2017, 2020; Bilella et al., 2016; Chometton et al., 2016). The posterior LHA contains the PSTN, the closely related small calbindin nucleus (CbN) and the Parvafox nucleus (Fig. 3), which receive inputs from insular and orbital areas, respectively (Tsumori et al., 2006; Chometton et al., 2016; Babalian et al., 2019; Barbier et al., 2020). From the Parvafox, orbital cortex projections continue and end in the Gemini nucleus (Babalian et al., 2019). Ventral medial prefrontal axons (i.e., from the infralimbic area) also innervate the caudal lateral LHA in rodents, but the exact distribution of these axons with regard to the posterior LHA nuclei still requires investigation. Ventral medial prefrontal axons also reach the PMd and enter the MBO (Shibata, 1989; Hurley et al., 1991; Gonzalo-Ruiz et al., 1992; Comoli et al., 2000; Fisk and Wyss, 2000). Therefore, the ventral medial prefrontal input is not limited to the posterior LHA. In contrast, dorsal medial prefrontal areas (cingulate) target the medial STN (Canteras et al., 1990; Parent and Hazrati, 1995a; Emmi et al., 2020).

#### The fornix system and the stria terminalis

Since the mammillary circuit (or Papez circuit) involves some major fiber tracts such as the fornix and the mammillothalamic tract, its general architecture was understood very early. It was known since the beginning of the 20th century that the origin of the fornix is the hippocampal formation (Cajal, 1909). However, Swanson and Cowan (Swanson and Cowan, 1977) and Meibach and Siegel (Meibach and Siegel, 1977) were the first to identify pyramidal neurons in the dorsal subiculum at the origin of the postcommissural fornix, while it was observed that Ammon’s horn projects mostly through the precommissural fornix to innervate the lateral septal complex (the lateral nucleus of the septum and the septofimbrial nucleus; Swanson et al., 1981). This was confirmed by many other authors (Shibata, 1989; van Groen and Wyss, 1990; Gonzalo-Ruiz et al., 1992), and it is now well established that the dorsal subiculum innervates the medial mammillary nucleus while the para-pre-postsubiculum innervates the lateral mammillary nucleus. The projections from these cortical areas reach the MBO through the fornix. By contrast, the projections from the ventral subiculum reach the hypothalamus through the medial cortico-hypothalamic tract (Canteras and Swanson, 1992a). In the anterior and postoptic hypothalamus, this tract courses parallel to the stria terminalis which arises in the amygdala, and both the medial cortico-hypothalamic tract and the stria terminalis converge and mostly end in the PMv. The stria terminalis carries, in part, glutamatergic axons from the posterior nucleus of the amygdala (Canteras et al., 1992a) which lies adjacent to the ventral subiculum and is a cortico-amygdalar nucleus with a pallial origin (Swanson and Petrovich, 1998). Therefore, the projections from the posterior amygdalar nucleus to the PMv should also be viewed as cortical in nature. Finally, and for the sake of completeness, other cortical nuclei of the amygdala (i.e., the anterior part of the basomedial nucleus) project through the direct amygdalo-hypothalamic pathway into the ventral posterior LHA (CbN; Barbier et al., 2017).

#### Conclusions about the connections between the cerebral cortex and the posterior hypothalamus

**Figure 4.**
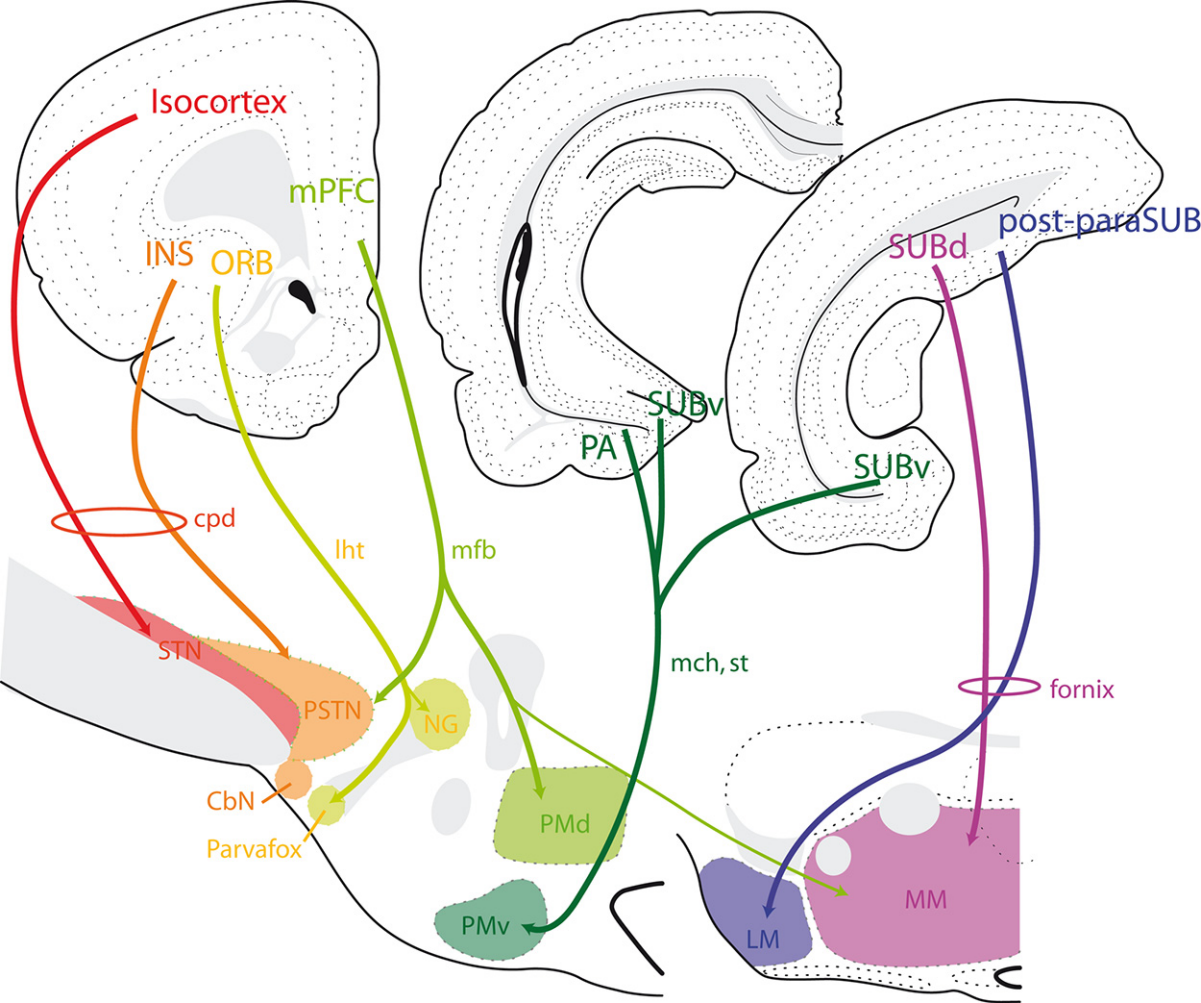
Line drawing illustrating the organization of cortical projections into the glutamatergic posterior hypothalamic nuclei. See text for details. cpd: cerebral peduncle; CbN: calbindin nucleus; INS: insular cortex; lht: lateral hypothalamic tract; LM: lateral mammillary nucleus; mch: medial cortico-hypothalamic tract; mfb: medial forebrain bundle; MM: medial mammillary nucleus; mPFC: medial prefrontal areas cortex; NG: nucleus gemini; ORB: orbital area cortex; PA: posterior nucleus of the amygdala; PMd: dorsal premammillary nucleus; PMv: ventral premammillary nucleus; post-paraSUB: posterior and parasubiculum; PSTN: para-STN; st: stria terminalis; STN: subthalamic nucleus; SUBd: dorsal subiculum; SUBv: ventral subiculum.

This short survey of the cortical innervation of the posterior hypothalamus shows that the glutamatergic nuclei of the posterior hypothalamus receive topographically organized inputs from the cortex, with the MBO and PMv receiving projections mostly from the allocortex (hippocampal formation, cortico-amygdala) and the STN receiving projections from the isocortex, while nuclei in-between these medial and lateral poles receive projections mostly from the periallocortex, including the ventral medial prefrontal, insular and orbital areas (Fig. 4). Therefore, the allocortical and periallocortical projections to the glutamatergic posterior hypothalamic structures are parallel to and topographically organized with the isocortical projections to the STN. In this way, the hyperdirect pathways arise from the cortical mantle as a whole and innervate glutamatergic nuclei of the posterior hypothalamic region. These cortical projections arise from pyramidal glutamatergic neurons. The STN is innervated by collaterals of descending axons that continue in the pyramidal tract. By contrast, the fornix ends in the MBO. However, at least in rats, the first axons constituting the fornix reach the mesencephalon during development and later emit collaterals that innervate the MBO while the distal mesencephalic branches recede (Stanfield et al., 1987).

### Subcortical afferences or indirect pathways

#### General organization of the subpallium

Based on the topographic organization of descending cortical inputs as well as on cytoarchitectural and neurochemical considerations, it has long been proposed that the cerebral nuclei of the basal telencephalon belong either to a striatal or to a pallidal compartment (Swanson, 2000, 2012; Risold, 2004). Therefore, the telencephalon would be organized according to a basic plan with the pallium innervating the striatum which itself projects onto the pallidum. This organization of the telencephalon has been adopted by the Allen Brain Institute (Allen Institute, 2004), whose atlases and databases are extensively used by the scientific community (Table 1). According to the Allen Brain Institute’s nomenclature, four striatal divisions receive projections from the cerebral cortex, including the dorsal striatum (caudoputamen) innervated by the isocortex as well as the ventral (nucleus accumbens, olfactory tubercle), medial (lateral septal complex) and caudal (striatal-like amygdalar nuclei) striatum receiving allocortical and periallocortical projections. The striatal compartment whose main cell type is the GABAergic somatospiny neuron, projects in a topographically organized way onto the dorsal [globus pallidus (GP)], ventral [ventral pallidum (VP), also named substantia innominate (SI)], medial (medial septal complex), and caudal [bed nucleus of the stria terminalis (BST)] pallidum (for additional information, see Tables 1, 2).

**Table 1:** Parcellation of the telencephalon

Cortical compartment	MO, Cing, …	mPFC, INS, SUBv	INS, cortico-AMY	SUB, CA
Striatal compartment	STRd (CPu)	STRv (Acb, FS, OT)	STRc (CEAc, CEAl, MEA)	STRm (LSN, SFN)
Pallidal compartment	PALd (GPe, GPi)	VP (SI)	PALc (BST, CEAm)	PALm (MSN, NDB)

Table summarizing the parcellation of the telencephalon based on the nomenclature of the Allen Brain Atlas and Swanson (Allen Institute, 2004; Swanson, 2004) with a slight modification from Barbier et al. (2020; CEAm is adjoined to the PALc, see comments in Table 2). Acb: accumbens nucleus; AMY: amygdala; BST: bed nucleus of the stria terminalis; CA: Ammon’s horn; CEAc: capsular part of the central nucleus of the amygdala; CEAl: lateral part of the central nucleus of the amygdala; CEAm: medial part of the central nucleus of the amygdala; Cing: cingulate cortex; CPu: caudoputamen; FS: fundus striatum; GPe: globus pallidus, external part; GPi: globus pallidus, internal part; INS: insular cortex; LSN: lateral septal nucleus; MEA: medial amygdalar nucleus; MO: somatomotor areas; MSN: medial septal nucleus; NDB: diagonal band nucleus; OT: olfactory tubercle; PALc: caudal pallidum; PALd: dorsal pallidum; PALm: medial pallidum; mPFC:; SFN: septofimbrial nucleus; SI: substantia innominata; STRc: caudal striatum; STRd: dorsal striatum; STRm: medial striatum; STRv: ventral striatum; SUB: subiculum; SUBv: ventral subiculum; VP: ventral pallidum.

**Table 2:** Origin of telencephalic subcortical inputs to the glutamatergic nuclei of the posterior hypothalamus

	Posterior hypothalamus						Vent MES	
	STN	PSTN/CbN	Pvfox/NG	PMv	PMd	MBO	SN	VTA
		
	(1, 2, 3, 4, 5)	(6, 7, 8, 9, 10, 11)	(4, 12, 13, 14)	(16, 17, 18, 19)	(15, 16)	(20, 21, 22, 23)	(24, 25, 26)	(4, 10, 19, 21, 26, 27, 28, 29, 30)
STRIATUM								
Dorsal striatum (caudoputamen)	+						++++	
Ventral striatum (nucleus accumbens, fundus of striatum, olfactory tubercle)		+		+			++	++++
Medial striatum (lateral septal nucleus)				+				++
Caudal striatum (central amygdalar nucleus, capsular and lateral parts^(^*^a^*^)^)		+					++	++
MEA^(^*^b^*^)^				++++^(^*^b^*^)^				+
PALLIDUM								
Dorsal pallidum (GP)	++++						+++	
VP ^(^*^c^*^)^	+++ central	++++ posterior	+++ anterior					+++
Medial pallidum			+++ NDB,MPO			++ MS		+
Caudal pallidum		++++ BSTrh, CEAm		++++ BSTpr	+++ BSTif		++	++

This table was realized based on the following references: (1) Canteras et al. (1990); (2) Graybiel et al. (1994); (3) Parent and Hazrati (1995a); (4) Groenewegen and Berendse (1990); (5) Groenewegen et al. (1993); (6) Barbier et al. (2020); (7) Barbier et al. (2017); (8) Chometton et al. (2016); (9) Dong and Swanson (2003); (10) Grove (1988); (11) Dong et al. (2001); (12) Price et al. (1991); (13) Heimer et al. (1990); (14) Gaykema et al. (1990); (15) Comoli et al. (2000); (16) Dong and Swanson (2004a,b); (17) Cavalcante et al. (2014); (18) Gu et al. (2003); (19) Risold and Swanson (1997); (20) Shibata (1989); (21) Swanson and Cowan (1979); (22) Vann (2010); (23) Vann and Aggleton (2004); (24) Gonzales and Chesselet (1990); (25) Gerfen and Bolam (2016); (26) Tomimoto et al. (1987); (27) Luo et al. (2011); (28) Geisler and Zahm (2005); (29) Kaufling et al. (2009); and (30) Phillipson (1979). Commentaries about the used parcellation: although we have remained very close to the nomenclature used by the Allen Brain Atlas (Allen Institute, 2004), a few adaptations seemed necessary to us. (a) The CEA is one of the striatal-like amygdalar nuclei. However, the original cytoarchitectonic study by McDonald (McDonald, 1982) revealed that only the lateral and central parts of the CEA contain striatal-like medium spiny neurons, while the medial part do not contain such neurons. The medial part of the CEA (CEAm) receives afferences from the lateral CEA as well as from the fundus striatum (belonging to the ventral striatum), which signify that the CEAm is targeted by striatal-like structures. Furthermore, it is intensely, selectively bidirectionally connected to the PSTN adjacent to the STN. Based on these considerations, the CEAm belongs to the pallidum and not to the striatum. This assertion is also compatible with developmental data (Bupesh et al., 2011; Barbier et al., 2020). (b) The MEA is also one of the striatal-like amygdalar nuclei. Without wishing to question this hypothesis, it is necessary to make a comment. Indeed, the MEA is made of a complex collection of neurons. In particular, it contains abundant populations of glutamatergic neurons with a hypothalamic or a pallial origin (Ruiz-Reig et al., 2018). These neurons are abundant in the posteroventral part of the medial amygdalar nucleus (MEApv) which sends dense projections to the PMv (Canteras et al., 1992b). Therefore, a better characterization of the neurochemical nature of the MEA projection to the PMv is necessary to understand the organization of this complex amygdalar nucleus. (c) For practical reason only, we divided the substantia innominata/VP into the three parts: the anterior VP is deep to the olfactory tubercle. The central anterior pallidum corresponds to most of the pallidum as illustrated by Root et al. (2015); the posterior VP corresponds to the posterior substantia innominata excluded from the VP by Root et al. (2015). BSTif: interfascicular part of the bed nucleus of the stria terminalis; BSTpr: principal part of the bed nucleus of the stria terminalis; BSTrh: rhomboid nucleus of the bed nucleus of the stria terminalis; CbN: calbindin nucleus; CEAm: medial part of the central nucleus of the amygdala; MBO: mammillary body; MPO: medial preoptic area; MS: medial septal nucleus; NDB: diagonal band nucleus; NG: nucleus gemini; PMd: dorsal premammillary nucleus; PMv: ventral premammillary nucleus; PSTN: para-STN; Pvfox: Parvafox nucleus; SN: substantia nigra; STN: subthalamic nucleus; VTA: ventral tegmental area.

#### The direct and indirect pathways of the basal ganglia

**Figure 5.**
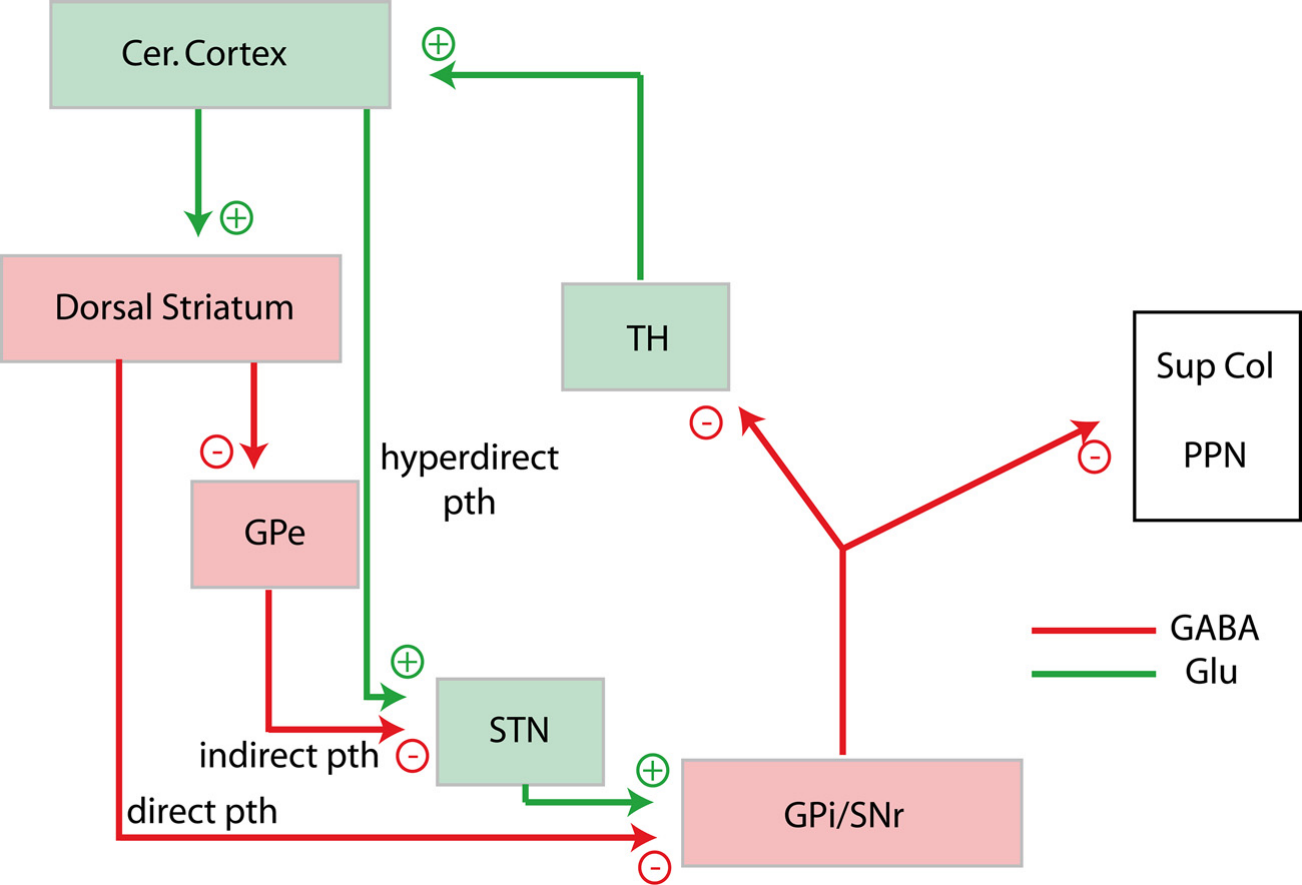
The basal ganglia network. Diagram summarizing the neurochemical organization of direct, indirect, and hyperdirect pathways of the basal ganglia network. GPi and SNr are considered to be the exit points of the network toward motor structures (superior colliculus and pedunculopontine nucleus). Cer. Cortex: cerebral cortex; Glu: glutamate; GPe: globus pallidus, external part; GPi: globus pallidus, internal part; PPN: pedunculopontine nucleus; pth: pathway; SNr: substantia nigra, reticular part; STN: subthalamic nucleus; Sup Col: superior colliculus; TH: thalamus.

Both striatal and pallidal compartments are then bidirectionally connected to the brainstem, but the organization of the descending pathways that connect these cerebral nuclei with the brainstem has been best portrayed for the dorsal striatum/dorsal pallidum, forming the well-known basal ganglia network (Fig. 5). Indeed, in addition to the hyperdirect pathway from the isocortex to the STN, the basal ganglia network is usually divided into direct and indirect pathways (Künzle, 1975; McGeorge and Faull, 1989; Graybiel et al., 1994; Parent and Hazrati, 1995a; Nambu et al., 2002; Graybiel, 2004; Gerfen and Bolam, 2016; Tecuapetla et al., 2016). The direct pathway involves several types of medium spiny neurons in the dorsal striatum that project in the internal part of the GP (GPi) and the reticular part of the SN (SNr). The indirect pathway originates from another class of medium spiny neurons of the dorsal striatum that project into the external part of the GP (GPe). The main output of the GPe is for the STN as well as for the SNr. In turn, the STN projects into the whole GP and the SNr. Therefore, the STN is an additional station between the striatum and GPi/SNr.

#### Organization of subcortical projections to the posterior hypothalamus

As hyperdirect-like projections were described for the glutamatergic nuclei of the posterior hypothalamus, the comparison with the STN can be prolonged by analyzing the origin of subcortical projections to other posterior nuclei of the hypothalamus. A general inspection of Table 2 that summarizes these data, reveals that the posterior hypothalamus is predominantly and intensely connected to the pallidal compartment of the telencephalon as defined by the Allen Brain Atlas Canteras et al. (1990); Graybiel et al. (1994); Parent and Hazrati (1995a); Groenewegen and Berendse (1990); Groenewegen et al. (1993); Barbier et al. (2020); Barbier et al. (2017); Chometton et al. (2016); Dong and Swanson (2003); Grove (1988); Dong et al. (2001); Price et al. (1991); Heimer et al. (1990); Gaykema et al. (1990); Comoli et al. (2000); Dong and Swanson (2004a,b); Cavalcante et al. (2014); Gu et al. (2003); Risold and Swanson (1997); Shibata (1989); Swanson and Cowan (1979); Vann (2010); Vann and Aggleton (2004); Gonzales and Chesselet (1990); Gerfen and Bolam (2016); Tomimoto et al. (1987); Luo et al. (2011); Geisler and Zahm (2005); Kaufling et al. (2009); and Phillipson (1979). By contrast, the striatal compartment is marginally connected to the posterior hypothalamus [a notable exception is the intense input from the medial amygdalar nucleus (MEA) to the PMv, but see the commentaries about the MEA in the comment (b) of Table 2; Ruiz-Reig et al. (2018)]. Resonating with the canonical direct pathway of the basal ganglia, the striatal compartment is intensely related to the SN/VTA.

**Figure 6.**
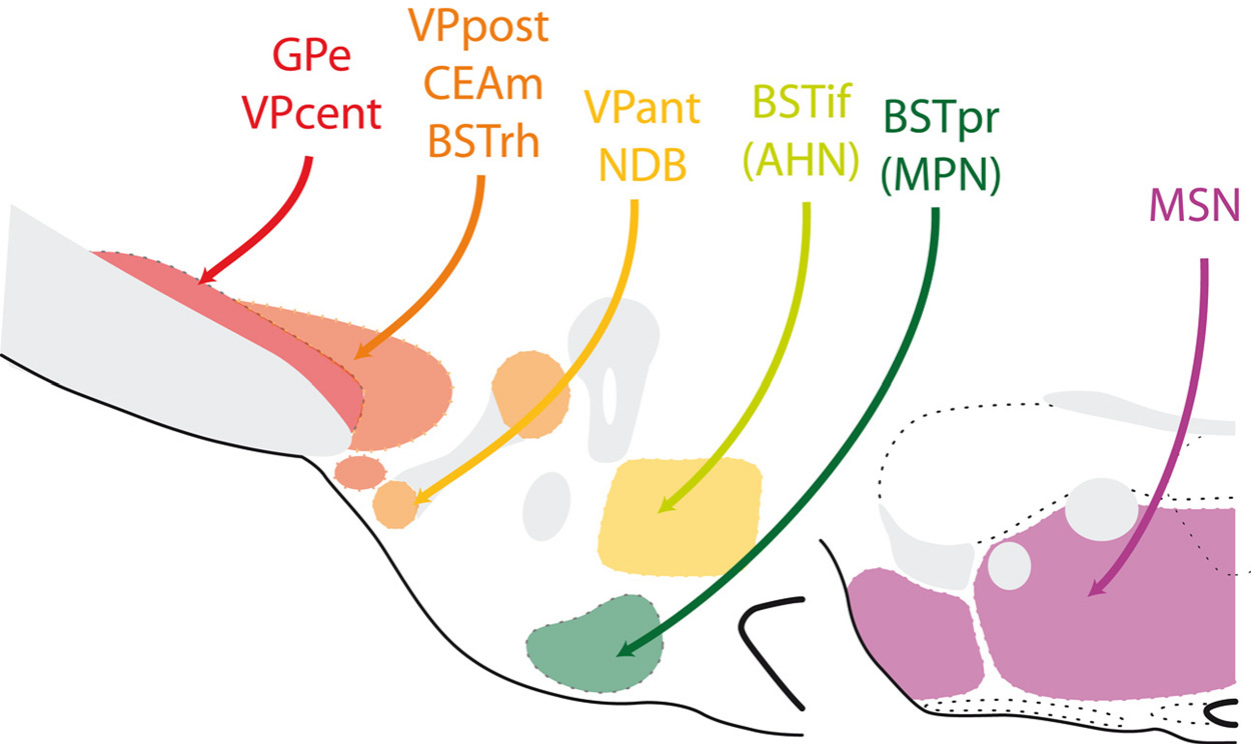
Subcortical input to the posterior hypothalamus. Line drawing to summarize the origin of the subcortical inputs to the glutamatergic nuclei of the posterior hypothalamus. See text for details. AHN: anterior hypothalamic nucleus; BSTif: interfascicular nucleus of the bed nuclei of the stria terminalis; BSTpr: principal nucleus of the BST; BSTrh: rhomboid nucleus of the BST; CEAm: medial part of the central nucleus of the amygdala; GPe: external part of the globus pallidus; MPN: medial preoptic nucleus; MSN: medial septal nucleus; NDB: nucleus of the diagonal band; VPant, cent, post: ventral pallidum, anterior, central, or posterior regions.

Projections from the pallidal compartment to the posterior hypothalamus are topographically organized (Fig. 6). Along the projection from the GPe to the whole STN, the medial tip of the STN receives inputs from the ventral VP (Groenewegen et al., 1993; Root et al., 2015; Groenewegen and Berendse, 1990). The rostral region of the VP (following the nomenclature of Root et al., 2015; see Table 2), sends its axons through the ventrolateral hypothalamic tract and innervates the Parvafox and Gemini nuclei (Lundberg, 1962; Heimer et al., 1990; Price et al., 1991). These nuclei also receive inputs from the magnocellular preoptic nucleus and from the nucleus of the diagonal band (Heimer et al., 1990; Groenewegen et al., 1993). The olfactory nature of this pathway was demonstrated by Price 30 years ago (Price et al., 1991). Located between the Parvafox and STN, the PSTN is targeted by posterior VP (Grove, 1988; Chometton et al., 2016). The PSTN also receives convergent inputs from the medial division of the central nucleus of the amygdala [CEAm; included in a recent study to the pallidal compartment, see the legend (a) of Table 2; Bupesh et al. (2011)], from the rhomboid nucleus of the BST and, to a lesser extent, from the anterolateral, and oval nuclei of the BST (Dong et al., 2001; Dong and Swanson, 2003, 2004a; Chometton et al., 2016; Barbier et al., 2017). The caudal BST projects mostly into the PMv and PMd. These two hypothalamic nuclei are innervated by projections from the principal (BSTpr) and interfascicular (BSTif) nuclei of the BST, respectively (Comoli et al., 2000; Gu et al., 2003; Dong and Swanson, 2004b; Cavalcante et al., 2014). Finally, the medial septal complex (medial pallidum) innervates the medial mammillary nucleus (Swanson and Cowan, 1979; Shibata, 1989; Vann and Aggleton, 2004; Vann, 2010). This input is not as dense as other pallidal projections into the posterior hypothalamic nuclei, but it is the sole subcortical projection from the telencephalon identified in the medial mammillary nucleus and it serves important functions in this nucleus (Dillingham et al., 2021).

**Figure 7.**
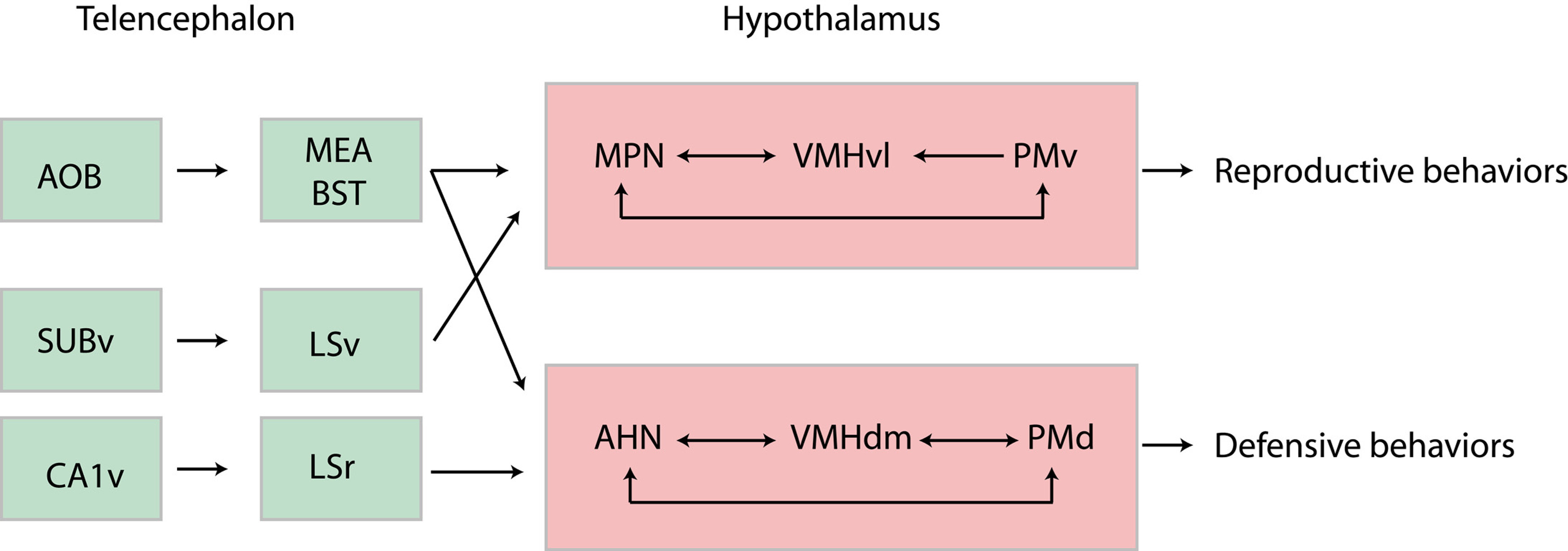
Hypothalamic circuits involving the PMd and PMv. PMd and PMv are embedded within intra hypothalamic circuits with other medial zone nuclei, including the MPN and AHN. These circuits are involved in reproductive and defensive behaviors. They are under the control of pheromonal informations from the AOB and MEA as well as from informations that originates in the ventral hippocampus (SUBv and CA1v). AHN: anterior hypothalamic nucleus; AOB: accessory olfactory bulb; BST: bed nucleus of the stria terminalis; CA1v: field CA1, Ammon’s horn ventral region; LSr: lateral septal nucleus, rostral part; LSv: lateral septal nucleus, ventral part; MEA: medial amygdalar nucleus; MPN: medial preoptic nucleus; PMd: dorsal premammillary nucleus; PMv: ventral premammillary nucleus; SUBv: ventral subiculum; VMHdm: ventromedial hypothalamic nucleus hypothalamus, dorsomedial part; VMHvl: ventromedial hypothalamic nucleus hypothalamus, ventrolateral part.

Both the PMv and the PMd are known to be integrated into circuits with other hypothalamic medial zone nuclei, and these circuits are also under the command of subcortical telencephalic projections (Fig. 7). The PMv is bidirectionally connected to the medial preoptic nucleus (MPN) while the PMd is bidirectionally linked with the anterior nucleus (AHN; Canteras et al., 1992b; Canteras and Swanson, 1992b; Risold et al., 1997; Swanson, 2000). The MPN shows a strong sexual dimorphism, and the MPN-PMv circuit is called the sexually dimorphic circuit (Simerly and Swanson, 1988; Canteras et al., 1992b; Swanson, 2000). The AHN and PMd are involved in defense responses (Canteras and Swanson, 1992b; Risold et al., 1994; Swanson, 2000). Both the MPN and the AHN receive strong inputs from the BSTpr and BSTif, respectively (Dong and Swanson, 2004b), along with intense innervation from the ventral and rostral parts of the lateral septal nucleus (LSNv and LSNr, respectively; Risold and Swanson, 1997).

## Functional Considerations

All glutamatergic nuclei of the posterior hypothalamus receive topographically arranged projections from the telencephalon which comprise inputs from the cortical mantle that are reminiscent of the hyperdirect pathway as well as from the pallidal compartment reminiscent of the indirect pathway. However, this whole analysis is worth considering only if it improves our understanding of the functional organization of this region. To date, most nuclei of the posterior hypothalamus have been studied independently and each one of them is involved in its own specific response: motor behavior for the STN, control of feeding for the PSTN/CbN, agonistic behaviors for the Parvafox, PMv, and PMd, and complex cognitive functions related to encoding spatial information for the MBO (Canteras and Swanson, 1992b; Parent and Hazrati, 1995a; Swanson, 2000; Gerfen and Bolam, 2016; Barbier et al., 2020; Dillingham et al., 2021). Therefore, no functional relationship seems to link these different structures, contrary to what the developmental and anatomic data suggest. To understand the functional organization of the glutamatergic posterior hypothalamic region as a whole, once again, the STN may serve as a model. Indeed, it is important to remember that we understand the functions of the STN in collaboration with and often as opposed to that of the striato-nigral direct pathway. Therefore, the function of each nucleus of the posterior hypothalamus should be considered within a larger anatomic network also involving the ventral mesencephalon. Indeed, the ventral mesencephalon is implicated in behavioral responses (motor, feeding, social, and agonistic behaviors) similar to those of the posterior nuclei of the hypothalamus (Wei et al., 2021).

### Summary of the functional organization of the basal ganglia network

At the lateral pole of the posterior hypothalamic glutamatergic region, STN functions are related to that of the basal ganglia network to which it belongs. GPi and SNr are the output stations of the basal ganglia network: they innervate the pedunculopontine nucleus and the superior colliculus that grant access to the somatic motoneurons and the cerebellar network (Gerfen and Bolam, 2016; Fig. 5). They also project into several nuclei of the thalamus forming the classic loops of the basal ganglia network with the motor cortex (Alexander et al., 1986; Parent and Hazrati, 1995b; Deniau et al., 1996; Haber, 2003; Kim and Hikosaka, 2015). However, as the medium spiny neurons in the caudoputamen as well as GP and SNr neurons are GABAergic, the direct pathway results in tonic inhibition of its targets which are disinhibited when cortical glutamatergic inputs stimulate the striatum and this pathway is also known as the “Go” pathway. On the other hand, the STN is glutamatergic and stimulates GPi and SNr neurons on disinhibition through the cortex-striatum-GPe pathway or activation by the hyperdirect pathway. Therefore, the activation of the STN through indirect or hyperdirect pathways, results in the inhibition of ongoing motor actions and the indirect pathway is also known as the “No-Go” pathway (Bahuguna et al., 2015; Baghdadi et al., 2017; Bariselli et al., 2019). This No-Go action is deemed important for the suppression of competing motor programs that would otherwise interfere with the execution of the desired movement, as well as for switching motor action and adapting behavior to environmental changes perceived by the isocortex (Wessel and Aron, 2013; Fife et al., 2017; Chen et al., 2020)

### Posterior hypothalamus and VTA functional networks

The VTA in the ventral mesencephalon is involved in similar behavioral responses to many nuclei of the posterior hypothalamus, excluding the STN and MBO. Through its connections with the accumbens nucleus and the VP, the VTA initiates approach or avoidance responses in relation to feeding or agonistic/social behaviors. Generally, the VTA is thought to be involved in reinforcing behavioral responses and increasing or decreasing reward-seeking behaviors (Bouarab et al., 2019; Morales and Margolis, 2017; Parker et al., 2019). Data that integrate posterior nuclei of the hypothalamus in the functional network of the VTA are lacking. An anterograde study illustrates projections from the PSTN into the VTA (Goto and Swanson, 2004). Unfortunately, the functional significance of these connections has not yet been further investigated. Nonetheless, anatomic links also exist through the ventral/caudal/medial striato-pallidal complexes or through other nuclei of the hypothalamus (Phillipson, 1979; Groenewegen et al., 1993; Risold and Swanson, 1997; Geisler and Zahm, 2005; Kaufling et al., 2009; Luo et al., 2011), suggesting at least indirect interactions at functional levels between the posterior hypothalamus and the VTA (Table 2).

### Social behaviors in relation to reproduction and parental care

**Figure 8.**
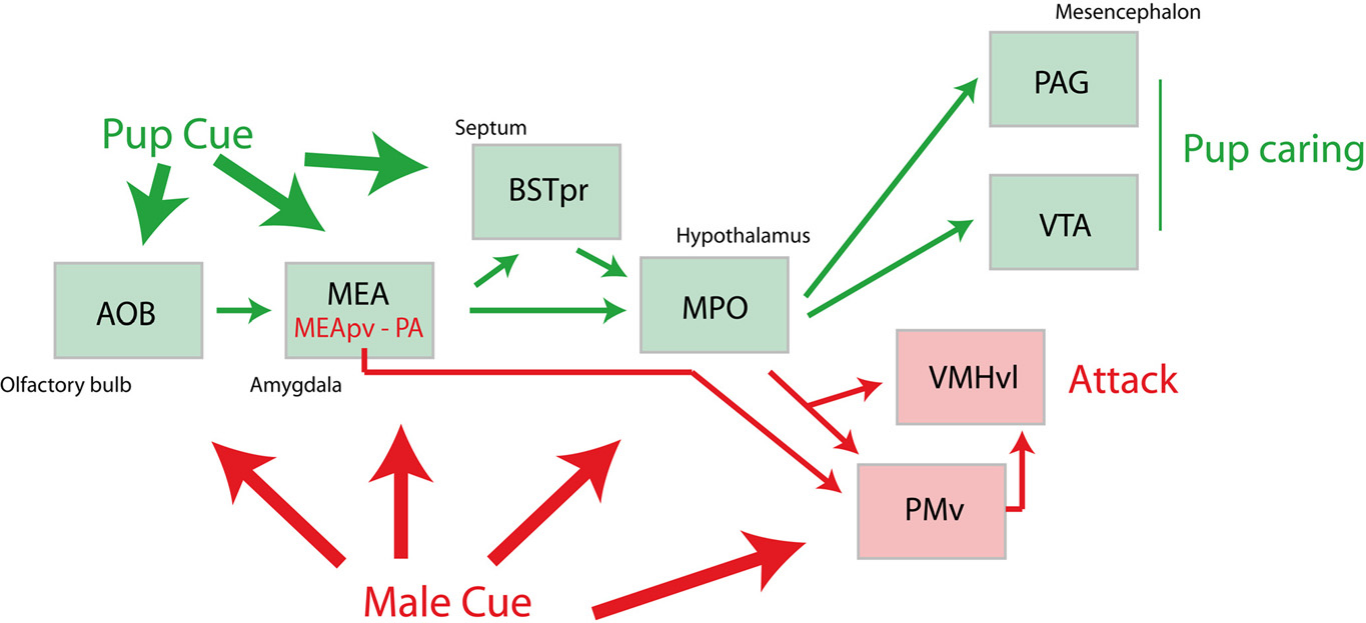
Circuit involved in pup caring and in conspecific attack. In lactating females, pheromonal and other pup cues are carried to the MPO. Projections from the MPO to the PAG and VTA are involved in pup approach and care. However, if a male approaches the pups, an attack reaction from the mother to protect the pups necessitates the PMv and VMHvl. The PMv and VMHvl also mediate intermale aggressions. Male cues are caried by MEApv and PA projections to the PMv. See Wei et al. (2021) for details. AOB: accessory olfactory bulb; BSTpr: principal nucleus of the bed nuclei of the stria terminalis; MEA: medial amygdalar nucleus; MEApv: medial amygdalar nucleus, posteroventral part; MPO: medial preoptic area; PA: posterior amygdalar nucleus; PAG: periaqueductal gray; PMv: ventral premammillary nucleus; VMHvl: ventromedial hypothalamic nucleus hypothalamus, ventrolateral part. VTA: ventral tegmental area.

The nucleus accumbens-VTA network is involved in reproduction through the regulation of sexual preferences (Beny-Shefer et al., 2017). The projections from the VTA to the nucleus accumbens can encode and predict key features of social interactions (Gunaydin et al., 2014). The medial preoptic area (MPO) is a key center for the expression of many aspects of reproductive behaviors. Several populations of neurons within this region serve distinct aspects of reproduction, including copulatory behaviors, nest building, pup retrieval and grooming. In lactating females, a specific medial preoptic-VTA pathway is involved in nursing and pup retrieval (Fang et al., 2018; Fig. 8). Moreover, oxytocinergic projections from the paraventricular nucleus of the hypothalamus to the VTA and SNc drive DAergic neuron activity in opposite directions by increasing the activity of the VTA and decreasing that of the SNc (Xiao et al., 2017). Oxytocin-modulated DAergic neurons give rise to canonical striatal projections and oxytocin release in the VTA is necessary to elicit social reward, and is involved in attachment or bonding between parents and pups.

The PMv is involved in many other aspects of reproductive behaviors as part of the sexually dimorphic circuit with the MPN: it receives pheromonal information from the MEA and BSTpr, and the exposure of individuals to conspecific pheromonal stimuli induces a strong c-Fos expression in the PMv (Yokosuka et al., 1999; Nordman et al., 2020). Then, depending on the hormonal status of the individual and the sex of the intruder, the PMv either facilitates copulation or promotes an aggressive response. For example, the PMv is involved in intermale aggression or male copulatory behavior (Pfaus and Heeb, 1997; Stagkourakis et al., 2018; Fig. 8). In the case of females in estrus, this nucleus stimulates lordosis behavior. This is also a key site for leptin’s regulation of reproduction, and it relays this information about the nutritional state to regulate gonadotropin-releasing hormone (GnRH) release (Leshan and Pfaff, 2014). In contrast to the VTA in lactating females, the PMv promotes a maternal aggressive response against a male intruder (Motta et al., 2013), but reports about the role of the PMv in caring for pups are lacking to date (Fig. 8; see also Wei et al., 2021).

Therefore, both the VTA and the PMv are connected to the medial preoptic region, but while the VTA plays a role in reinforcing social bonds between partners and parents/infants, the role of the PMv is dictated by the hormonal status of the individual and the sex and status of conspecifics, and its role ranges from copulatory behavior to fight initiation, depending of context.

### Feeding behavior

The VTA through a rewarding action involving the nucleus accumbens, promotes the ingestion of hedonic food (Valdivia et al., 2014; Coccurello and Maccarrone, 2018; Koch et al., 2020). In general, DA-deficient mice are hypoactive, aphagic and adipsic (Zhou and Palmiter, 1995). The virally-induced rescue of DAergic signaling in the ventral striatum selectively restores the feeding of DA-deficient mice (Szczypka et al., 1999). Therefore, DAergic projections from the VTA to the ventral striatum, affect the motivation to eat regardless of homeostatic constraints.

By contrast, the PSTN and CbN have been implicated in the cognitive and physio-pathologic control of feeding (Barbier et al., 2020). Some authors also considered the PSTN as part of a satiety network (Zséli et al., 2016). These nuclei respond to the ingestion of hedonic food and to sickness. The response to hedonic food ingestion is even stronger if this food is consumed for the first time (Chometton et al., 2016; Barbier et al., 2020). However, they are preferentially involved in limiting food consumption in a way that was compared with the No-Go action of the STN (Barbier et al., 2020). The network involving these nuclei encompasses bidirectional connections with the insular cortex, the CEA and the posterior SI. Additionally, it comprises ascending calcitonin gene-related peptide (CGRP) inputs from the parabrachial nucleus in the pons that convey aversive signals from the periphery (Carter et al., 2015; Chometton et al., 2016; Barbier et al., 2017, 2020; Chen et al., 2018; Palmiter, 2018).

Therefore, both the PSTN/CbN and the VTA respond to hedonic food intake, but DAergic signaling in the VTA increases consumption while the PSTN/CbN limits the ingestion of such food if circumstances are not favorable (e.g., neophobia, sickness).

### Defensive behavior

Both the VTA and the PMd have been extensively involved in the response to environmental threats. These responses include freezing, escape and even fighting. Concerning the VTA, it has been shown that noxious stimuli are able to excite ventral DAergic neurons while dorsal DAergic neurons are inhibited (Brischoux et al., 2009). DAergic inputs in the basolateral nucleus of the amygdala mediate the freezing response in contextual conditioned fear (de Oliveira et al., 2017) and, more recently, Barbano et al. (2020) identified a population of Vglut2-VTA neurons that mediate escape responses to threatening stimuli.

The PMd has also long been associated with a defense circuit involving connections with the AHN in the anterior hypothalamus, the ventral part of the anteromedial nucleus of the thalamus, and the dorsolateral sector of the periaqueductal gray (Blanchard et al., 2003; Aguiar and Guimarães, 2011; Litvin et al., 2014). This nucleus also depends on olfactory/pheromonal inputs for its functions. Initially, it was mostly involved in freezing responses to either a predator or predator odors, or to a dominant conspecific (social threat; Canteras et al., 1992b, 2008, 2015; Pavesi et al., 2011; Rangel et al., 2018). Anatomical evidence for a circuit suggesting that the AHN and PMd may influence eye and head movements was described long ago (Risold and Swanson, 1995). Indeed, recently, a study by Wang et al., provided further insights into the function of the PMd (Wang et al., 2021). These authors showed that this nucleus coordinates escape with spatial navigation. Projections from the PMd to the dorsolateral periaqueductal gray are necessary for the flight response, but its projection into the ventral part of the anteromedial nucleus of the thalamus is required to choose complex and suitable routes to escape a threat. Therefore, this nucleus plays a specific role in versatile context-specific escape.

### Mammillary nuclei cooperation with the basal ganglia network

The MBO forms the medial pole of the glutamatergic posterior hypothalamic region. It is made of two nuclei that have similar and parallel projections with the ventral or dorsal tegmental nuclei of Gudden and with the anterior thalamic nuclei, but have distinct cell types and functions (Vann and Aggleton, 2004; Vann, 2010). Being the farthest from the STN, these two nuclei have no obvious connections with the ventral mesencephalon. Nevertheless, the current notion concerning the functions of these nuclei suggests that they may complete or influence basal ganglia action in the expression of behavior.

#### Occulomotor and head direction

Eye and head movements are important for scanning the environment and their control is indissociable from attentional processes and the ability to adapt to the environment.

The basal ganglia direct and indirect pathways play a key role in many aspects of these processes through the projections from the SNr to the superior colliculus (Kim et al., 2017; Hikosaka et al., 2019). By and large, the basal ganglia control gaze, gaze orientation and smooth pursuit (saccadic eye movements). Again, direct and indirect pathways play complementary roles with the indirect pathway being important for object choice and deteriorating gaze orientation to “bad” objects (Kim et al., 2017; Hikosaka et al., 2019). In addition, deep-brain stimulation of the STN used for the treatment of Parkinson’s disease, affects eye movements (Klarendic and Kaski, 2021). Other striatal compartments may as well affect oculomotor responses from the SN. The amygdalo (from the CEA, caudal striatum)-nigral pathway is involved in boosting oculomotor action in motivating situations (Maeda et al., 2020).

Projections from the superior colliculus into the pontine nucleus are important to control basal ganglia oculomotor responses. Indeed this nucleus along with the nucleus reticularis tegmenti pontis are intimately involved in the visual guidance of eye movements and are known to influence the cerebellar vermis and flocculus (Allen and Hopkins, 1990; Liu and Mihailoff, 1999). Interestingly, the descending output of the MBO into the nucleus reticularis tegmenti pontis and the dorsomedial pontine nucleus are also well documented (Allen and Hopkins, 1990; Liu and Mihailoff, 1999). Therefore, the MBO may also mediate visual and vestibular related information through an anatomic pathway that includes mammillopontine projections to these precerebellar relay nuclei.

**Figure 9.**
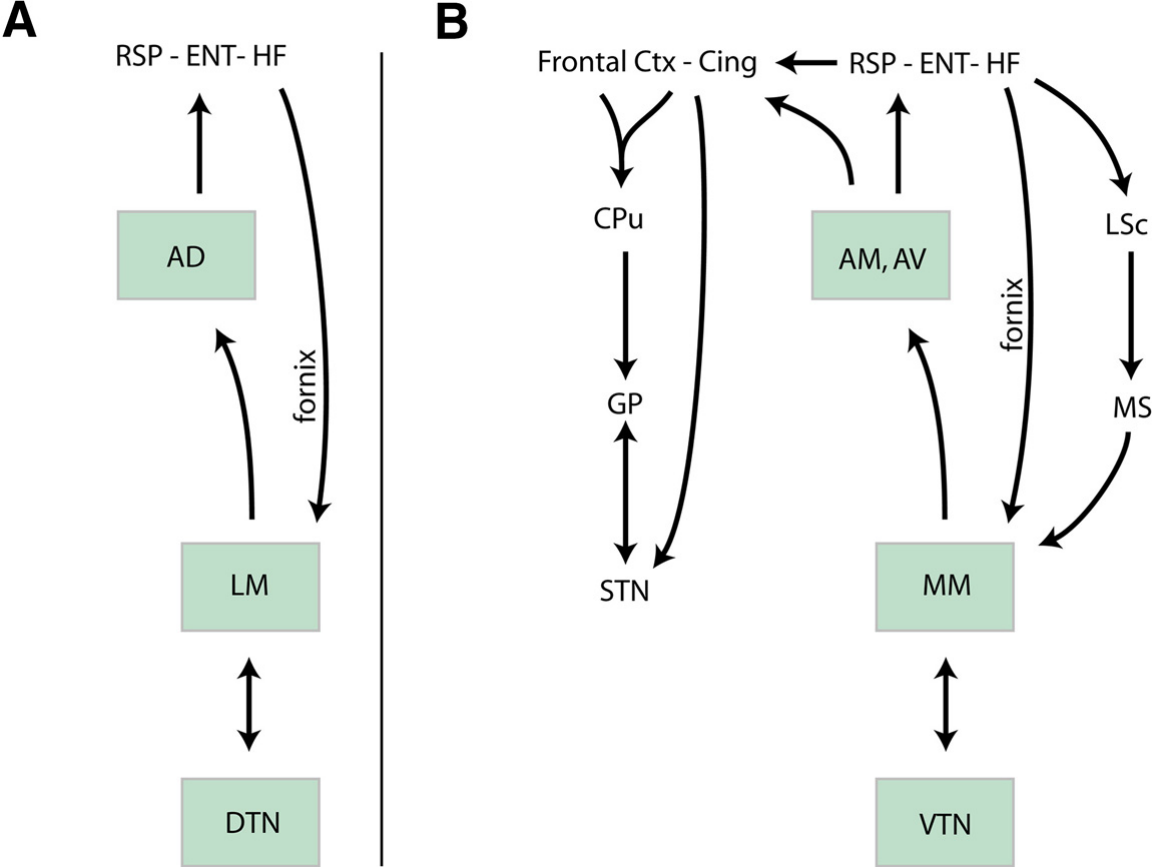
Organization of circuits involving the LM and MM. ***A***, The LM is bidirectionally connected to the DTN. It also projects into the AD of the anterior thalamus which innervates the RSP and hippocampal formation. In turn the LM is innervated by the fornix. This circuit is involved in head direction. ***B***, The MM is bidirectionally connected with the VTN and projects into the AM and AV of the anterior thalamus. The AV innervates the RSP, ENT, and HF, but through the AM, MM can also influence frontal areas and the anterior cingulate cortex, and modulates, along hippocampal projections, the activity of indirect and hyperdirect pathways from these isocortical areas (for more details, see text and Dillingham et al., 2021). AD: anterodorsal nucleus of the thalamus; AM: anteromedial nucleus of the thalamus; AV: anteroventral nucleus of the thalamus; Cing: cingulate cortex; CPu: caudoputamen; Ctx: cortex; DTN: dorsal tegmental nucleus (Gudden); ENT: entorhinal area; GP: globus pallidus; HF: hippocampal formation; LM: lateral mammillary nucleus; LSc: lateral septal nucleus, caudal part; MM: medial mammillary nucleus; MS: medial septal nucleus; RSP: retrosplenial area; STN: subthalamic nucleus; VTN: ventral tegmental nucleus (Gudden).

However, the lateral mammillary nucleus (LM) is mainly concerned with head direction. The LM along with the dorsal tegmental nucleus of Gudden, is probably particularly important for transforming vestibular information to signal head direction. Head direction cells are found in the LM but also in all the structures belonging to the LM circuit including the Gudden’s dorsal tegmental nucleus, anterodorsal nucleus of the thalamus, retrosplenial cortex and postsubiculum (Vann and Aggleton, 2004; Vann, 2010; Fig. 9). Selective LM lesions abolish the anterior thalamic head direction signal as well as the directional specificity of hippocampal place field repetition. Head direction cells are critical for navigation and recent computational and experimental studies show that they interact with place and grid cells in large parts of the temporal cerebral cortex to support spatial memory, scene construction, novelty detection and mental navigation (Bicanski and Burgess, 2018; Soman et al., 2018; LaChance et al., 2020).

#### Medial mammillary nucleus and theta rhythm

Theta band oscillations encode information critical to mnemonic processing across a wide range of diencephalic and cortical brain areas, including the hippocampal formation, medial septum, MBO, Gudden’s ventral tegmental nucleus (VTN) and anterior nuclei of the thalamus (ATN; Vann and Aggleton, 2004; Vann, 2010; Dillingham et al., 2021). Over the years, theta activity in the medial mammillary nucleus (MM) was thought to depend on descending input from the dorsal hippocampus through the fornix, but recent data indicate that MM-VTN interactions comprise an independent theta source and that the MBO-ATN pathway forms a medial diencephalic theta network that arises independently of the hippocampus (Dillingham et al., 2021). Therefore, the mammillothalamic pathway may contribute to contextual encoding, and as suggested by Dillingham and colleagues, “the MB-ATN axis may be specifically tuned (via theta oscillations) to process and relay context-rich and time-critical information that is further integrated and distributed to higher-order areas by thalamocortical circuits.”

At this point, it is important to remember that functional connectivity between basal ganglia neuronal activity and theta band activity in the hippocampus exists (Allers et al., 2002). The medial prefrontal cortex (MPF) is affected by theta rhythm generated in the hippocampus (Colgin, 2011). These connections are important for decision-making, as a dorsal medial prefrontal-subthalamic pathway supports action selection in a spatial working memory task (Heikenfeld et al., 2020) and theta oscillations in the STN also increase when individuals are making decisions in the presence of conflict (Zaghloul et al., 2012; Zavala et al., 2013, 2018). A next step would be to verify whether the MM-ATN pathway could also be involved in such responses and whether a coupling of functions between the MM and STN occurs through an MM-ATN-MPF-STN pathway that is inferred by anatomy (Fig. 9).

### Concluding functional considerations

Glutamatergic posterior hypothalamic structures are involved in controlling basal ganglia motor output or in strategic decision-making regarding reactions toward conspecifics, ingestion of hedonic food or finding a path to escape a threat. As a whole, they appear to perform non-rewarding actions correlated to spatial or internal contexts, while the SN/VTA is associated with reinforcement, motivation and reward of actions also relying on gaze and attention. However, the medial and lateral nuclei of the posterior hypothalamus show differences in the kind of responses in which they are involved: the STN, PSTN, and PMv are clearly involved in controlling specific motor/behavioral outputs by directly or indirectly interacting with the telencephalic basal nuclei/ventral mesencephalic networks. The MBO influences cognitive processes through ascending thalamo-cortical projections and interacts with the medial wall of the pallium and of the striatum/pallidum whose functions are less dependent on ascending DAergic mesencephalic inputs. In particular, the MM contribute to the perception of the spatiotemporal context by the hippocampal formation which then provides this information to the iso/periallocortex. The PMd has an interesting intermediary position.

Active research related to the role of the STN within the basal ganglia network is constantly being conducted in human and animal models (Hikosaka et al., 2019). To date, similar studies that examine the comparative roles of the posterior hypothalamic networks and that of the SN/VTA are still rare but will constitute a promising future field of research.

## Hypothesis and Perspectives

**Figure 10.**
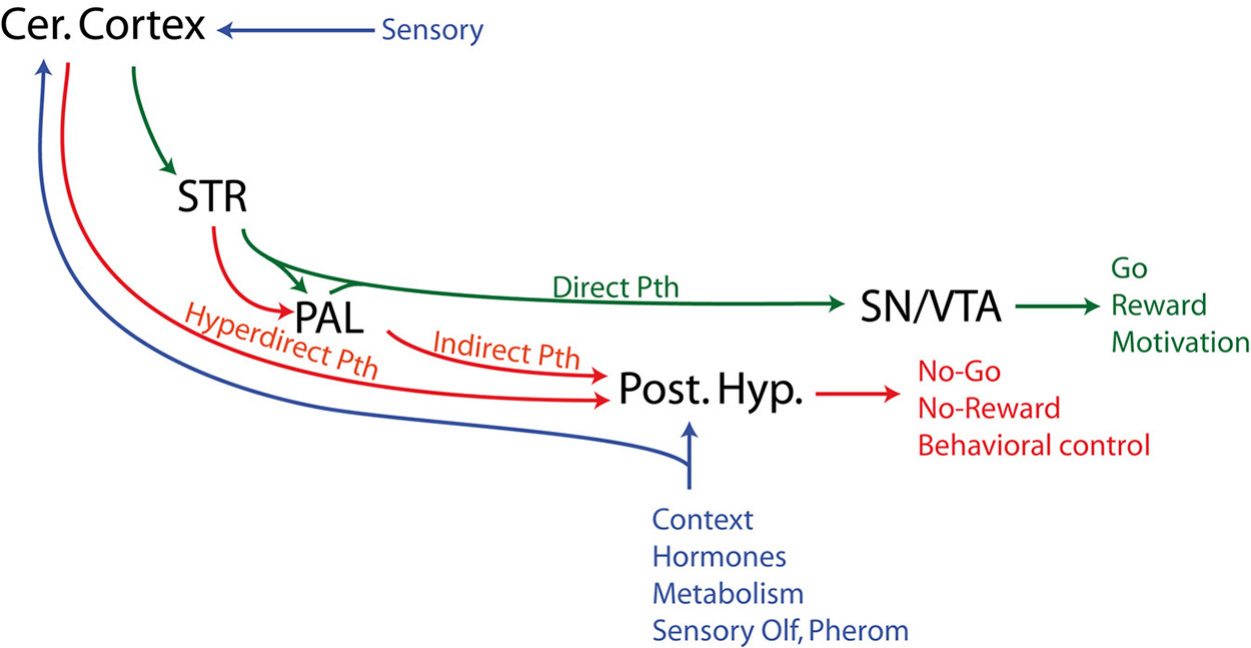
Diagram summarizing the organization of the telencephalic input to the glutamatergic posterior hypothalamus and SN/VTA. The posterior hypothalamus receives convergent cortical and pallidal afferences while the SN/VTA receives striatal inputs. The GPe input to the SNr is not illustrated to keep the schema simple and as they were not addressed within this paper. Cer. Cortex: cerebral cortex; PAL: pallidum; Post. Hyp.: posterior hypothalamus; Pth: pathway; SN: substantia nigra; STR: striatum; VTA: ventral tegmental area.

A little more than two decades ago, it was established that the circuits involving the allocortex and periallocortex, cerebral nuclei and medial zone nuclei of the hypothalamus resembled in terms of their structures to the basal ganglia loop with the isocortex. In the meantime, it was noticed that the STN, which is an essential component of the basal ganglia network, belonged to the hypothalamus. To reconcile the two observations, we have reviewed recent developmental, anatomic and functional data concerning the STN and the posterior hypothalamus. The developmental data showed that the STN is integrated within a larger glutamatergic posterior hypothalamic region generated in a specific embryonic anlage that is adjacent to the ventral mesencephalon where the SN/VTA differentiates. We then realized that this posterior hypothalamic region receives convergent and topographically organized cortical and pallidal projections. This pattern of telencephalic input can be compared with the intense striatal projections that reach the SN/VTA (Fig. 10). Finally, the structures belonging to this posterior glutamatergic hypothalamic region and the SN/VTA serve complementary functions to organize behaviors. In the end, it becomes tempting to hypothesize here that the glutamatergic posterior hypothalamic region is involved in decision-making processes in situations that are dictated by environmental or internal contexts and that require immediate behavioral adaptation (e.g., social or predator threats), or by bypassing the direct pathways of the basal ganglia to limit the pursuit of rewarding actions and prevent negative consequences (e.g., limit the ingestion of palatable but unknown food).

Baed on this analysis, it is plausible to hypothesize that hypothalamic longitudinal circuits that interconnect hypothalamic medial zone nuclei and the basal ganglia circuitry are built on a similar basic plan (see also Croizier et al., 2015). The fact that the STN has a hypothalamic origin is a clear evidence supporting this hypothesis. The relationship between the preoptic region and the pallidal anlage in the embryonic brain is another sign that should not be neglected. Pursuing investigations in this direction (see as well Swanson et al., 2019) may prove to be fruitful to achieve a better understanding of how the hypothalamus is integrated within large scale neural circuits in the prosencephalon.

## Synthesis

Reviewing Editor: Nathalie Ginovart, University of Geneva Decisions are customarily a result of the Reviewing Editor and the peer reviewers coming together and discussing their recommendations until a consensus is reached. When revisions are invited, a fact-based synthesis statement explaining their decision and outlining what is needed to prepare a revision will be listed below. The following reviewer(s) agreed to reveal their identity: Gonzalo Alvarez-Bolado, Alessandro Bilella.

Both reviewers agreed that the manuscript is timely and provides important insights into the functional organization of the posterior hypothalamus, integrating the subthalamic nucleus as a hypothalamic nucleus, and, as such, support the publication. However, both reviewers also thought that the manuscript cannot be accepted in its present form. Parts of the manuscript are not abundantly clear, which detract from the impact of the work. There was thus a consensus that the paper should be revised to be made more understandable to non-specialists and to improve its readability.

Please find the specific comments and queries raised by the reviewers below:

Reviewer 1 :

This is a review attempting to demonstrate 1) that the subthalamic nucleus, STN, should be considered part of the posterior hypothalamus (not the basal ganglia), and 2) that this “neuroanatomical rearrangement” would go a long way to explain the structural basis of <<decision-making (...) in situations (...) that require (...) to limit the pursuit of rewarding actions and prevent negative consequences>>. The authors are successful in both pursuits; they are however less successful in conveying their argument (which is technically correct) in an accessible way to a wide readership.

The present work is a key contribution to ongoing efforts to answer the ages-old question: what is the general organization (plan) of the brain? It focuses on one of the pieces of the puzzle, the STN, and, after considering its development and connections, makes a convincing argument to relocate it in the posterior hypothalamus, PH. The authors show how in this way the novel STN-PH would have a behavioral “no-go” function (i.e. “do not pursue reward”) working as a counterweight to the “go” function of the striato-nigral-ventral tegmental area (SN/VTA) pathway.

The authors review the literature expertly and extensively and use clear figures to make their point.

In the last fifty years or so, increasingly more accurate axon-tracing as well as behavioral techniques have given a vast amount of information about the structure and function of the brain. Efforts to put this all together in a cogent circuit diagram of the brain have started to give fruit since the beginning of this century and have accelerated substantially in the last few years.

Unfortunately, although the culmination of more than a century of efforts by neuroanatomists is of general interest to all neuroscientists, the authors of this particular contribution write as if they were speaking to just a few specialists. The text comes across as particularly impenetrable and the importance of the subject and the suitability of the arguments are not powerfully highlighted. This is a very welcome contribution and deserves to be published after a formal makeover for readability.

Reviewer 2 :

This review attempts at summarizing the current findings that support a hypothalamic origin of the subthalamic nucleus (STN), in contrast to the well-established theory that the STN is part of the subthalamus. In particular, they review anatomy, transcription factors, neurogenesis and development of the STN and compare them with the hypothalamus anatomy and development.

The review is interesting and timely, however, it needs careful English proofreading and further editing to be accepted for publication.

Here some comments the authors should address in their revised version:

1. As mentioned before, the manuscript is quite chaotic and disorganized. The authors may want to add subheadings in each paragraph to increase readability.

2. In chapter 3.1, this sentence “From the Parvafox, projections running through the ventrolateral hypothalamic tract end in the nucleus Gemini” is incorrect. In Babalian et al., 2019, the projections are not from the Parvafox nucleus but they originate from the LO and VLO nuclei. Please revise.

3. To strengthen the conclusion that STN is part of the hypothalamus, the authors may want to consider adding also data from electrophysiology of STN in relation with the rest of the brain and compare it with the hypothalamic ones.

4. Also, in the final paragraph 6., the authors could suggest further experiments still missing in the field, that may be performed to support the idea of the hypothalamic origin of STN.
